# SMART HOME TECHNOLOGY FOR OLDER ADULTS WITH MOBILITY DISABILITIES: POTENTIAL AND CHALLENGES

**DOI:** 10.1093/geroni/igz038.3217

**Published:** 2019-11-08

**Authors:** Kenneth A Blocker, Lyndsie M Koon, Travis Kadylak, Widya A Ramadhani, Roshanak Khaleghi, Christopher Kovac, Ramavarapu S Sreenivas, Wendy A Rogers

**Affiliations:** 1 University of Illinois at Urbana-Champaign, Champaign, Illinois, United States; 2 University of Illinois, Urbana-Champaign, Illinois, United States

## Abstract

Recently, there has been a significant expansion in the number of smart and connected technologies for assisting individuals with a variety of tasks within the home. Examples include digital home assistants (e.g., Amazon Echo), smart lights, smart plugs, robotic vacuums, as well as a multitude of other devices. Such technologies hold the potential to support independence for older adults with long-term mobility disabilities, as they may experience challenges engaging in daily activities. The aim of the current study was to utilize a comprehensive approach with an interdisciplinary team to improve understanding of how to integrate smart technology into older adults’ homes. We focused on identifying functionality that would be useful to them, understanding their perceptions, and developing instructional support. We conducted interviews among older adults with, and without, long-term mobility disabilities to better understand their attitudes towards digital assistants, identify needs for instructional support, and test the usability of our instructional protocol. The overall goal of this research is to improve understanding of older adults’ perceptions of these technologies and identify usability challenges within the home. The instructional protocol offers support by reducing the identified barriers to initial adoption and continued use to promote aging-in-place and improving overall quality of life for older adults with long-term mobility disabilities.

